# Can Early Rehabilitation after Total Hip Arthroplasty Reduce Its Major Complications and Medical Expenses? Report from a Nationally Representative Cohort

**DOI:** 10.1155/2015/641958

**Published:** 2015-06-04

**Authors:** Daniel Chiung-Jui Su, Kuo-Shu Yuan, Shih-Feng Weng, Rong-Bin Hong, Ming-Ping Wu, Hing-Man Wu, Willy Chou

**Affiliations:** ^1^Department of Physical Medicine and Rehabilitation, Chi-Mei Medical Center, Tainan, Taiwan; ^2^Department of Business Management, National Sun Yat-sen University, Taiwan; ^3^Department of Medical Research, Chi-Mei Medical Center, Tainan, Taiwan; ^4^Division of Urogynecology, Department of Obstetrics and Gynecology, Chi Mei Medical Center, Tainan, Taiwan; ^5^Department of Recreation and Health-Care Management and Institute of Recreation Industry Management, Chia Nan University of Pharmacy and Science, Taiwan

## Abstract

*Objective*. To investigate whether early rehabilitation reduces the occurrence of posttotal hip arthroplasty (THA) complications, adverse events, and medical expenses within one postoperative year. *Method*. We retrospectively retrieve data from Taiwan's National Health Insurance Research Database. Patients who had undergone THA during the period from 1998 to 2010 were recruited, matched for propensity scores, and divided into 2 groups: early rehabilitation (Early Rehab) and delayed rehabilitation (Delayed Rehab). *Results*. Eight hundred twenty of 999 THA patients given early rehabilitation treatments were matched to 205 of 233 THA patients given delayed rehabilitation treatments. The Delayed Rehab group had significantly (all *p* < 0.001) higher medical and rehabilitation expenses and more outpatient department (OPD) visits than the Early Rehab group. In addition, the Delayed Rehab group was associated with more prosthetic infection (odds ratio (OR): 3.152; 95% confidence interval (CI): 1.211–8.203; *p* < 0.05) than the Early Rehab group. *Conclusions*. Early rehabilitation can significantly reduce the incidence of prosthetic infection, total rehabilitation expense, total medical expenses, and number of OPD visits within the first year after THA.

## 1. Introduction

A disabled hip joint is a major inconvenience because it reduces one's functional ability and secondarily increases comorbidities caused by immobility. In severe cases, a total hip arthroplasty (THA) is mandated. One review [1] showed that primary osteoarthritis (OA) is the main indication for more than 65% of all primary THA performed in the USA, Scandinavia, Scotland, and Australia. Lai et al. [2] also reported that the most common three diagnoses for THA in Taiwan were avascular necrosis (AN) (46.9%), OA (41.6%), and femoral neck fracture (1.5%).

The success of THA is its predictable pain relief, improvements in quality of life, and restoration of normal function [3]. Brander et al. [4] also point out that, to achieve maximal functional performance, rehabilitation should focus on reducing pain, increasing range of motion, and strengthening the hip muscles, for example, the gluteals and quadriceps and the hamstring muscles.

To achieve better outcomes after joint replacement, recent consensus statements have advocated research on the timing of rehabilitation intervention. Chen et al. [5] reported that early rehabilitation after total knee arthroplasty (TKA) is associated with reducing major complications such as deep vein thrombosis (DVT) and prosthetic infection. Trampuz and Zimmerli [6] also found that prosthetic infection is associated with poor skin and soft-tissue healing, which is secondary to poor circulation that can be improved through rehabilitation. In addition, according to Anderson Jr. et al. [7], rehabilitation can be used as a mechanical prophylactic against DVT. But the question remains whether early rehabilitation after total hip arthroplasty can bring more benefit to the patients in the aspect of reducing complications and medical utilization in comparison to delayed rehabilitation.

In Taiwan, there is still no routine rehabilitation intervention after THA. There are a few protocols taken from Brotzman's* Clinical Orthopaedic Rehabilitation* textbook [8]. There is, however, no strong evidence that suggests how to decide when rehabilitation intervention is appropriate and whether different rehabilitation intervention timings affect the outcome of THA. In Brotzman's protocol, therapeutic exercises should begin on the first postoperative day and consist of lower extremity isometrics (gluteals, quadriceps, and hamstring) and ankle pumps. From the second to the fifth postoperative day, passive or active range-of-motion exercises of the hip within allowed ranges, heel slides (heel toward buttocks), sitting heel raises, and large arc quads should be gradually added. One week postoperatively, standing hip flexion to 90 degrees, hip extension, and hip abduction of the surgically repaired leg should be also done.

In addition, Husby et al. [9] point out that early maximal strength training 1 week postoperatively is a feasible and an efficient treatment for regaining muscular strength for patients who have undergone THA.

To the best of our knowledge, there is no large scale study focus on the proper time of rehabilitation intervention following THA and how the different timing of rehabilitation may impact on patient's outcome including medical expenses in the long run. We wanted to clarify whether early post-THA rehabilitation intervention of rehabilitation attenuates complications and comorbidities. We also hypothesized that early post-THA rehabilitation intervention reduces the need for postoperative medical services and the number of outpatient visits.

## 2. Methods

### 2.1. Data Source

Our data were obtained from Taiwan's National Health Insurance Research Database (NHIRD), which is maintained by the National Health Research Institutes (NHRI) specifically for research. It is an administrative database that contains all medical care claims for outpatient, inpatient, and emergency room services of all NHI patients, which is approximately 99.5% of Taiwan's 23 million people. The data we used are a representative sample of the NHIRD, which contains all original claim data (International Classification of Diseases, 9th revision, Clinical Modification (ICD-9-CM)), medical expenditures, rehabilitation expenditures, treatment during admission and after discharge, prescriptions, hospital levels, and each enrollee's age and gender of 1 million people randomly sampled from the 23 million beneficiaries in the NHIRD. No significant differences exist in the age, gender, or insured amount distributions between patients in our data and the original NHIRD with *p* value = 0.187 [10]. The NHIRD has been used by many researchers [11] for dozens of published studies. The Institutional Review Board (IRB) of Chi Mei Foundation Hospital approved this study and waived the requirement of informed consent because the datasets in the NHIRD have no identifiable personal information.

### 2.2. Study Design

**Figure 1:**
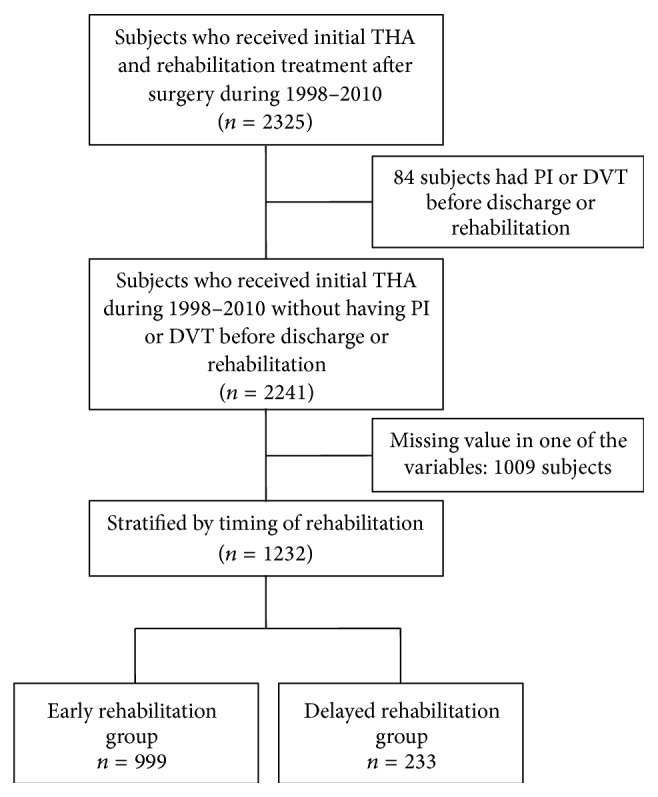
Flowchart of subjects selection and assignment. THA: total hip arthroplasty; PI: prosthetic infection; DVT: deep venous thrombosis.

We identified 2325 patients who had been discharged from their initial THA (ICD-9-CM procedure code: 81.51) and had undergone rehabilitation within the first postoperative year between January 1998 and December 2010. Exclusion criteria included predischarge prosthetic infection (PI) or DVT (84 patients); this was done to minimize the risk of over- or underestimating medical expenditures. Another 1009 patients were excluded because they were missing data for one of the studied variables. We finally enrolled 1232 patients in the study (Figure 1).

We then subgrouped the patients, based on when they began rehabilitation (treatment codes: 42001–42016, 43001–43008, and 43026), into the Early Rehab (within 1 week after discharge; *n* = 999) and Delayed Rehab (1 week or more after discharge; *n* = 233) groups. The comorbidities looked at in this study had to be present before the date of the initial THA; they were osteoarthritis (OA) (ICD-9-CM: 715.15, 715.25, and 715.35); avascular necrosis (AN) (ICD-9-CM: 733.34); hypertension (HTN) (ICD-9-CM: 401-405); diabetes mellitus (DM) (ICD-9-CM: 250); and poor renal function (PRF) (ICD-9-CM: 585).

### 2.3. Outcome Measures

Prosthetic infection (PI) (ICD-9-CM: 996.66), deep vein thrombosis (DVT) (ICD-9-CM: 453), and revision of hip arthroplasty (RHA) (ICD-9-CM procedure code: 81.53) within 1 year after discharge were used as the primary outcome measures. We also recorded, as one of the outcome measures, the number of visits to the OPD (regardless of the reason for the visit) within the first year after being discharged with a diagnosis of THA. Medical expenses, including total medical expenses and expenses for rehabilitation exclusively, were calculated for the first postdischarge year.

### 2.4. Statistical Analysis

Initial comparisons of baseline demographic and clinical characteristics for the early and delayed rehabilitation groups were made using Pearson *χ* ^2^ tests for categorical variables and independent sample *t*-tests for continuous variables. Because the Early and Delayed Rehab groups may differ substantially in a number of ways, propensity-score matching was used to reduce the selection bias in our hypothesis: many confounding covariates may be present in an observational study with this number of variables. Score matching identified the predicted probability of obtaining 1 Early Rehab patient versus 4 Delayed Rehab patients from the logistic regression model based on gender, age, length of stay, Charlson Comorbidity Score (CCS), trauma code, and the comorbidities of OA, AN, HTN, DM, and PRF.

Moreover, based on propensity-score matching, a linear regression model was used to examine how the different timing of rehabilitation influenced total medical expenses, total rehabilitation expenses, and the number of OPD visits while controlling for gender, age, group, length of stay, CCS, trauma code, and comorbidity. Finally, a logistic regression model was used to assess the risk of post-THA-associated complications (PI, DVT, and RHA) for the two rehabilitation groups, after controlling for the same confounding variables. All of the analyses were performed using SAS 9.3.1 for Windows (SAS Institute, Cary, NC, USA). Significance was set at *p* < 0.05 (2-tailed).

## 3. Results

Based on propensity matching, 820 of 999 THA patients given early rehabilitation treatments matched to 205 of 233 THA patients given delayed rehabilitation treatments. Both groups were well balanced, after they had been given a propensity score, in these demographic and clinical variables: gender, age, age group, length of stay, OPD visits, total medical expenses, total rehabilitation expenses, CCS, E code, and a series of comorbidities (OA, HTN, DM, PRF, PI, DVT, and RHA) (Table 1).

Linear regression analyses showed that delayed rehabilitation group had higher total medical expenses (*p* < 0.001), higher total rehabilitation expenses (*p* < 0.001), and more postoperative OPD visits (*p* < 0.001) than the early rehabilitation groups (Table 2).

Logistic regression analyses showed that the delayed rehabilitation group was associated with a higher rate of prosthetic infection (odds ratio (OR): 3.152; 95% confidence interval (CI): 1.211–8.203; *p* < 0.05) when compared with early rehabilitation group. There was no significant difference between the incidence rates of DVT (OR: 1.309; 95% CI: 0.212–8.072; *p* > 0.050) or revision of hip replacement (OR: 2.346; 95% CI: 0.825–6.675; *p* > 0.050) between the early and delayed rehabilitation groups (Table 3).

The power of the primary outcome (prosthetic infection) of this study was more than 0.95, calculated using software Gpower for logistic regression with odds ratio = 3.152, prosthetic infection rate under null hypothesis is 0.039, and alpha error probability = 0.05, assuming the distribution of dependent variable was binomial with a balanced design (*p* = 0.5) with equal sample frequencies. Moreover, the power of the significant outcome (total medical expenses, total rehabilitation expenses, and OPD visits) of this study was also more than 0.95.

## 4. Discussion

An increasing number of hip replacements are performed each year throughout the world [12, 13]. To achieve better outcomes after joint replacement, recent consensus statements have advocated research on the timing of rehabilitation intervention. Not only is post-THA rehabilitation highly important, a recent meta-analysis of randomized controlled trials emphasized that exercise-based interventions before THA can reduce pain and improve physical function for people awaiting hip replacement surgery [14]. Currently, however, there is no consensus about how soon rehabilitation should start after the THA and what benefits it might bring to the patients.

In clinical practice, rehabilitation methods after THA can include hip-joint mobilization, using low-resistance weights to strengthen the surrounding muscles, gait training, and early maximal strength training [9]. One study [9] reported that early maximal strength training beginning 1 week postoperatively is feasible and an efficient treatment to regain muscular strength for patients who have undergone THA. Another one [15] reported that rehabilitation emphasizing weight bearing and postural stability might be advisable 4 months or more after surgery.

Typically, however, orthopedic surgeons will have second thoughts about early rehabilitation after THA for fear that early weight bearing, especially in uncemented THA, will loosen the prosthesis. In response to this, recent studies [16, 17] have reported that full weight bearing immediately after uncemented THA has no adverse effects. One prospective randomized study [18] reported no adverse effects and no significant differences in stem migration of the acetabular component during the first 6 postoperative weeks, 3 months, and one year in patients who engaged in immediate weight bearing after uncemented THA.

We found that when rehabilitation was initiated within the first week after discharge, PI during the first postoperative year was lower than that for rehabilitation initiated later. The real reason for the decreased PI rate needs further investigation. Regardless of factors such as a surgeon's technique or sterile preparation before and during the operation, which is beyond the frame of this study, prosthetic infection is associated with poor skin and soft-tissue healing, which is secondary to poor circulating problems [6, 19]. Early rehabilitation might promote the circulation around the replaced hip and therefore reduce the PI rate.

Secondly, another outcome measure in our study is DVT. In clinical practice, both pharmacological and mechanical DVT prophylaxes are possible. Routine thromboprophylaxis using aspirin, warfarin, or low-molecular-weight heparin, however, is associated with morbidity [20, 21]. With mechanical prophylaxis, rehabilitation such as simple leg lifts, elevating the foot off the bed, isotonic and isometric exercises, and active and passive ankle motion can be used. Although mechanical prophylaxis is recommended for early rehabilitation [7, 22], we found no significant difference in how much pharmacological and mechanical methods lowered the rate of DVT. One reason might be that the incidence of DVT in the Asian population is relatively low [23, 24], which makes it difficult to determine the efficacy of early rehabilitation for lowering the incidence of DVT [25–27]. Another is that a recent review [28] reported continuing controversy about early physiotherapy for thromboprophylaxis, and its effect has not yet been supported by level I/II evidence.

Most THA failures that occur within the first 2 years and require RHA can be attributed to joint instability (33%) and infection (24%) [29]. Moreover, aseptic loosening was the cause of approximately 18% of revision at less than 2 years after THA. The incidence of failed THA increases to over 90% 10 or more years after replacement [30]. With early rehabilitation, joint instability can be improved by strengthening the weak abductor muscles of the hip and lowering the rate of dislocation [30]. We found no significant difference between early and delayed rehabilitation groups, which indicated that RHA is multifaceted and more factors should be considered deciding to do the surgery.

Thirdly, we found that when comparing the total medical expenses or the total rehabilitation expenses in the first year after THA, the costs for the early rehabilitation group were much less than those for the delayed rehabilitation group. In our study, total medical expense included the patient's total medical expense for the first year after THA, which can be used to interpret the patient's general medical condition after THA, that there is no significant difference in the CCS or comorbidities between the two study groups. This result is especially important now because most of the health insurance policies around the world emphasize the cost efficiency of each therapy and have implemented policies such as diagnosis-related groups (DRGs) [31]. In Taiwan, the Bureau of National Health Insurance (BNHI) pays for THA procedures under DRG but also offers a Clinical Pathway (CP) to ensure the quality of THA. However, because in DRGs the BNHI does not require rehabilitation after THA, rehabilitation becomes an option in CP and causes orthopedic surgeons to rarely consult physiatrists for rehabilitation intervention in the first place. Our study carries important message to the health insurance companies that early rehabilitation is ultimately more cost-efficient.

Finally, Taiwan's NHI does not restrict accessibility to medical services or frequency of use. Moreover, because medical copayments are relatively low in Taiwan, patients are free to choose where and when to seek medical help [32]. Medical care is widely available to all of Taiwan's people. Therefore, it is the policy of the BNHI and lack of coordination between physicians that lead to delayed or absent rehabilitation intervention.

*Study Limitations*. This study has several limitations. Firstly, it used NHIRD claims data for all analyses; these data are used primarily for administrative rather than clinical purposes. Therefore, detailed clinical information is often lacking in the database: the clinical presentation of the patients, the content of rehabilitation they underwent, the surgeons' techniques, and the exact surgical approach used, for example, anterolateral or posterolateral, and cemented or cementless THA. Secondly, we investigated whether early rehabilitation had a positive impact on medical expense and complications. Because of the limitations of the information in the NHIRD, we cannot know the exact date of the operation or what rehabilitation the patient underwent between admission and discharge. Thus, we can only set the time as, for example, “one week after discharge” as the cut-point and compare outcome differences. This shortcoming suggests that, in the future, prospective studies to verify the association between the timing of rehabilitation and post-THA prognosis are needed.

## 5. Conclusion

The present study sends an important message to health policy makers around the world that intentional cost cutting might cause adverse events for patients and significantly increase postoperative expenditures. We found that early rehabilitation significantly reduced the rate of prosthetic infection, total rehabilitation costs, total medical expenses, and the number of THA-related OPD visits during the first year after a THA.

## Figures and Tables

**Table 1:** Descriptive statistics among patients receiving total hip arthroplasty.

	Variables					
	Before propensity score			After propensity score		
	Early RG	Delayed RG	*p* value	Early RG	Delayed RG	*p* value
Total, *n* (%)	999 (81.09)	233 (18.91)		820 (80.00)	205 (20.00)	
Gender, *n* (%)						
Female	434 (43.44)	106 (45.49)	0.5701	355 (43.29)	94 (45.85)	0.5086
Male	565 (56.56)	127 (54.51)		465 (56.71)	111 (54.15)	
Age, mean ± SD	56.48 ± 14.71	59.80 ± 14.63	0.0019	56.19 ± 13.63	57.48 ± 13.83	0.2270
Age group						
<65	681 (68.17)	136 (58.37)	0.0044	583 (71.10)	134 (65.37)	0.1094
≧65	318 (31.83)	97 (41.63)		237 (28.90)	71 (64.63)	
Length of stay, mean ± SD	8.66 ± 5.53	8.70 ± 5.30	0.9152	8.52 ± 5.17	8.64 ± 5.37	0.7644
OPD visits, mean ± SD	28.75 ± 21.55	41.49 ± 29.20	<0.0001	28.54 ± 21.67	39.20 ± 27.70	<0.0001
Total medical expenses (USD/year), mean ± SD	77002 ± 151086	123911 ± 189473	0.0005	70788 ± 128596	119293 ± 194903	0.0008
Total rehabilitation expenses (USD/year), mean ± SD	2630 ± 9490.3	11002 ± 19588.5	<0.0001	2794 ± 10013	10278 ± 17701	<0.0001
CCS, mean ± SD	0.71 ± 1.35	0.88 ± 1.39	0.0915	0.60 ± 1.15	0.67 ± 1.09	0.4257
E code patients, *n* (%)	49 (4.90)	13 (5.58)	0.6715	35 (4.27)	10 (4.88)	0.7031
*Complications *						
PI, *n* (%)	14 (1.40)	10 (4.29)	0.0040	11 (1.34)	8 (3.90)	0.0150
DVT, *n* (%)	5 (0.50)	2 (0.86)	0.5128^a^	5 (0.61)	2 (0.98)	0.5694^a^
RHA, *n* (%)	13 (1.30)	8 (3.43)	0.0236	10 (1.22)	6 (2.93)	0.0778
OA, *n* (%)	530 (53.05)	134 (57.51)	0.2190	440 (53.66)	113 (55.12)	0.7069
AN, *n* (%)	467 (46.75)	98 (42.06)	0.1961	391 (47.68)	92 (44.88)	0.4718
HTN, *n* (%)	239 (23.92)	74 (31.76)	0.0134	163 (19.88)	48 (23.41)	0.2626
DM, *n* (%)	84 (8.41)	20 (8.58)	0.9309	73 (8.90)	20 (9.76)	0.7035
PRF, *n* (%)	19 (1.90)	3 (1.29)	0.7832^a^	14 (1.71)	3 (1.46)	0.8068

*Note*. ^a^Fisher's exact test. RG: rehabilitation group; OPD: outpatient department; CCS: Charlson Comorbidity Scores; PI: prosthetic infection; DVT: deep vein thrombosis; RHA: revision of hip arthroplasty; OA: osteoarthritis; AN: avascular necrosis; HTN: hypertension; DM: diabetes mellitus; PRF: poor renal function.

**Table 2:** The linear regression modeling of total medical expenses within one year among propensity-score matched patients.

	Outcome variables								
	Total medical expenses			Total rehabilitation expenses			OPD visits		
	*β*	SE	*p* value	*β*	SE	*p* value	*β*	SE	*p* value
Timing of rehabilitation									
Delayed RG versus early RG	47853	10301	<0.0001	7253	927	<0.0001	9.57	1.68	<0.0001
Gender (male versus female)	16782	9055	0.0641	1442	815	0.0771	−3.15	1.48	0.0033
Age group (<65 versus ≧65)	7134	9520	0.4539	1369	857	0.1106	6.44	1.55	<0.0001
Length of stay	4788	827	<0.0001	53	74	0.4752	0.20	0.14	0.1448
CCI score	18029	4107	<0.0001	472	370	0.2019	3.74	0.67	<0.0001
E code patient	−22563	21223	0.2880	3345	1911	0.0803	0.86	3.46	0.8041
*Complications *									
OA	−19882	11187	0.0758	−1007	1007	0.3177	−0.91	1.83	0.6195
AN	−16269	11243	0.1482	−2228	1012	0.0279	−2.30	1.83	0.2096
HTN	−12337	10699	0.2491	1825	963	0.0585	7.77	1.75	<0.0001
DM	6184	16587	0.7094	1215	1493	0.4159	5.58	2.71	0.0395
PRF	345490	32959	<0.0001	−2364	2967	0.4258	8.15	5.38	0.1299
Constant	22827	14793	0.1231	1873	1332	0.1600	23.90	2.41	<0.0001

*Note.* RG: rehabilitation group; OPD: outpatient department; CCS: Charlson Comorbidity Scores; PI: prosthetic infection; DVT: deep vein thrombosis; RHA: revision of hip arthroplasty; OA: osteoarthritis; AN: avascular necrosis; HTN: hypertension; DM: diabetes mellitus; PRF: poor renal function.

**Table 3:** The logistic regression modeling on prosthetic infection (PI), deep vein thrombosis (DVT), and revision of hip arthroplasty (RHA) within one year after THA.

	Outcome variables								
	PI			DVT			RHA		
	OR	95% CI	*p* value	OR	95% CI	*p* value	OR	95% CI	*p* value
Timing of rehabilitation									
Delayed versus early	3.152	1.211–8.203	0.0187	1.309	0.212–8.072	0.7720	2.346	0.825–6.675	0.1100
Gender (male versus female)	4.118	1.230–13.78	0.0217	1.366	0.285–6.547	0.6964	1.456	0.483–4.393	0.5044
Age group (<65 versus ≧65)	1.130	0.395–3.229	0.8194	0.635	0.110–3.648	0.6105	0.768	0.244–2.415	0.6513
Length of stay	0.970	0.885–1.063	0.5123	1.018	0.871–1.190	0.8220	1.030	0.957–1.108	0.4355
CCS	1.062	0.711–1.588	0.7684	2.440	1.152–5.172	0.0199	0.998	0.610–1.633	0.9945
E code patient (yes versus no)	9.873	2.881–33.840	0.0003				3.486	0.643–18.911	0.1478
*Complications *									
OA (yes versus no)	1.917	0.604–6.083	0.2693				3.153	0.833–11.935	0.0908
AN (yes versus no)	0.989	0.316–3.097	0.9854				1.554	0.451–5.352	0.4847
HTN (yes versus no)	1.015	0.283–3.639	0.9815	0.828	0.088–7.805	0.8692	0.670	0.169–2.662	0.5692
DM (yes versus no)	1.466	0.306–7.016	0.6322	0.230	0.009–5.961	0.3759	3.944	0.896–17.368	0.0696
PRF (yes versus no)	3.405	0.298–38.921	0.3242						

*Note.* CCS: Charlson Comorbidity Scores; OR: odds ratio; OA: osteoarthritis; AN: avascular necrosis; HTN: hypertension; DM: diabetes mellitus; PRF: poor renal function.

## References

[1] Merx H., Dreinhöfer K., Schräder P. (2003). International variation in hip replacement rates. *Annals of the Rheumatic Diseases*.

[2] Lai Y. S., Wei H. W., Cheng C. K. (2008). Incidence of hip replacement among national health insurance enrollees in Taiwan. *Journal of Orthopaedic Surgery and Research*.

[3] Brown T. E., Cui Q., Mihalko W. M. (2009). *Arthritis and Arthroplasty: The Hip*.

[4] Brander V. A., Stulberg S. D., Chang R. W. (1994). Rehabilitation following hip and knee arthroplasty. *Physical Medicine & Rehabilitation Clinics of North America*.

[5] Chen H.-W., Chen H.-M., Wang Y.-C., Chen P.-Y., Chien C.-W. (2012). Association between rehabilitation timing and major complications of total knee arthroplasty. *Journal of Rehabilitation Medicine*.

[6] Trampuz A., Zimmerli W. (2005). Prosthetic joint infections: update in diagnosis and treatment. *Swiss Medical Weekly*.

[7] Anderson F. A., Hirsh J., White K., Fitzgerald R. H. (2003). Temporal trends in prevention of venous thromboembolism following primary total hip or knee arthroplasty 1996–2001: findings from the Hip and Knee Registry. *Chest*.

[8] Brotzman S. B., Manske R. C. (2011). *Clinical Orthopaedic Rehabilitation: An Evidence-Based Approach*.

[9] Husby V. S., Helgerud J., Bjørgen S., Husby O. S., Benum P., Hoff J. (2009). Early maximal strength training is an efficient treatment for patients operated with total hip arthroplasty. *Archives of Physical Medicine and Rehabilitation*.

[10] http://w3.nhri.org.tw/nhird//en/Data_Subsets.html#S3.

[11] Wu M.-P., Weng S.-F., Hsu Y.-W., Wang J.-J., Kuo H.-C. (2013). Medical attendance for lower urinary tract symptoms is associated with subsequent increased risk of outpatient visits and hospitalizations based on a nationwide population-based database. *PLoS ONE*.

[12] Hirvonen J., Blom M., Tuominen U. (2006). Health-related quality of life in patients waiting for major joint replacement. A comparison between patients and population controls. *Health and Quality of Life Outcomes*.

[13] Brownlow H. C., Benjamin S., Andrew J. G., Kay P. (2001). Disability and mental health of patients waiting for total hip replacement. *Annals of the Royal College of Surgeons of England*.

[14] Gill S. D., McBurney H. (2013). Does exercise reduce pain and improve physical function before hip or knee replacement surgery? A systematic review and meta-analysis of randomized controlled trials. *Archives of Physical Medicine and Rehabilitation*.

[15] Trudelle-Jackson E., Emerson R., Smith S. (2002). Outcomes of total hip arthroplasty: a study of patients one year postsurgery. *Journal of Orthopaedic and Sports Physical Therapy*.

[16] Bodén H., Adolphson P. (2004). No adverse effects of early weight bearing after uncemented total hip arthroplasty: a randomized study of 20 patients. *Acta Orthopaedica Scandinavica*.

[17] Kishida Y., Sugano N., Sakai T. (2001). Full weight-bearing after cementless total hip arthroplasty. *International Orthopaedics*.

[18] Thien T. M., Ahnfelt L., Eriksson M., Strömberg C., Kärrholm J. (2007). Immediate weight bearing after uncemented total hip arthroplasty with an anteverted stem: a prospective randomized comparison using radiostereometry. *Acta Orthopaedica*.

[19] Powers K. A., Terpenning M. S., Voice R. A., Kauffman C. A. (1990). Prosthetic joint infections in the elderly. *American Journal of Medicine*.

[20] Coventry M. B., Nolan D. R., Beckenbaugh R. D. (1973). ‘Delayed’ prophylactic anticoagulation: a study of results and complications in 2,012 total hip arthroplasties. *The Journal of Bone & Joint Surgery—American Volume*.

[21] Patterson B. M., Marchand R., Ranawat C. (1989). Complications of heparin therapy after total joint arthroplasty. *The Journal of Bone and Joint Surgery—American Volume*.

[22] Kolbach D. N., Sandbrink M. W., Hamulyak K., Neumann H. A., Prins M. H. (2004). Non-pharmaceutical measures for prevention of post-thrombotic syndrome. *Cochrane Database of Systematic Reviews*.

[23] Kim Y.-H., Suh J.-S. (1988). Low incidence of deep-vein thrombosis after cementless total hip replacement. *Journal of Bone and Joint Surgery—Series A*.

[24] Warwick D., Williams M. H., Bannister G. C. (1995). Death and thromboembolic disease after total hip replacement. A series of 1162 cases with no routine chemical prophylaxis. *The Journal of Bone and Joint Surgery—British Volume*.

[25] Wong K. L., Daguman R., Lim K. H., Shen L., Lingaraj K. (2011). Incidence of deep vein thrombosis following total hip arthroplasty: a Doppler ultrasonographic study. *Journal of Orthopaedic Surgery*.

[26] Sudo A., Sano T., Horikawa K., Yamakawa T., Shi D., Uchida A. (2003). The incidence of deep vein thrombosis after hip and knee arthroplasties in Japanese patients: a prospective study. *Journal of Orthopaedic Surgery*.

[27] Kim Y.-H., Oh S.-H., Kim J.-S. (2003). Incidence and natural history of deep-vein thrombosis after total hip arthroplasty. *The Journal of Bone and Joint Surgery—British Volume*.

[28] Kurmis A. P. (2010). Review article: thromboprophylaxis after total hip replacement. *Journal of Orthopaedic Surgery*.

[29] Ulrich S. D., Seyler T. M., Bennett D. (2008). Total hip arthroplasties: what are the reasons for revision?. *International Orthopaedics*.

[30] Werner B. C., Brown T. E. (2012). Instability after total hip arthroplasty. *World Journal of Orthopaedics*.

[31] Beaty L. (2005). Understanding diagnostic related groups (DRGs) and inpatient hospital reimbursement. *Gastroenterology Nursing*.

[32] Haung C.-Y., Wang S.-P., Chiang C.-W. (2010). Cost feasibility of A pre-checking medical tourism system for U.S. patients undertaking joint replacement surgery in Taiwan. *Chang Gung Medical Journal*.

